# Mouse embryo phenotyping with optical coherence tomography

**DOI:** 10.3389/fcell.2022.1000237

**Published:** 2022-09-09

**Authors:** Deirdre M. Scully, Irina V. Larina

**Affiliations:** Department of Integrative Physiology, Baylor College of Medicine, Houston, TX, United States

**Keywords:** mouse, embryo, development, optical coherence tomography, *in vivo* imaging, heart

## Abstract

With the explosion of gene editing tools in recent years, there has been a much greater demand for mouse embryo phenotyping, and traditional methods such as histology and histochemistry experienced a methodological renaissance as they became the principal tools for phenotyping. However, it is important to explore alternative phenotyping options to maximize time and resources and implement volumetric structural analysis for enhanced investigation of phenotypes. Cardiovascular phenotyping, in particular, is important to perform *in vivo* due to the dramatic structural and functional changes that occur in heart development over relatively short periods of time. Optical coherence tomography (OCT) is one of the most exciting advanced imaging techniques emerging within the field of developmental biology, and this review provides a summary of how it is currently being implemented in mouse embryo investigations and phenotyping. This review aims to provide an understanding of the approaches used in optical coherence tomography and how they can be applied in embryology and developmental biology, with the overall aim of bridging the gap between biology and technology.

## Introduction

The mouse embryo is an important mammalian model for basic genetic research because it is genetically similar to humans and has a completely mapped genome which is highly versatile for genetic manipulation (Dietrich et al., 1996; Gridley and Murray, 2022). Advances in genetic engineering have led to a plethora of genetically manipulated mouse models which are invaluable for enhancing our understanding of human developmental diseases (Moon, 2006; Leduc et al., 2017), but as a result, the market for mouse mutants of human diseases is saturated, and inefficient phenotyping tools cannot accommodate the demand (Cacheiro et al., 2019). Advanced imaging methods, which facilitate high-throughput phenotyping, will be critical to ensure that the field of developmental biology can capitalize on large-scale, genome-wide screens for new models of human disease. In this way, advanced imaging in embryology has the potential to unlock unprecedented insights into the origins of congenital abnormalities (Dickinson et al., 2016).

Traditional structural phenotyping is carried out by histological and histochemical methods which are disadvantaged by their destructive nature, inability to capture live structure and dynamics, and the total loss of three-dimensional information (Hillman, 2000). Volumetric imaging techniques represent an exciting avenue of exploration for embryologists and developmental biologists (Wang et al., 2020). The most common non-destructive imaging modalities for phenotyping whole mouse embryos include ultrasound biomicroscopy (UBM) and micro-magnetic resonance imaging (µMRI), both of which have remarkable capabilities for live, *in utero* imaging (Phoon and Turnbull, 2003; Parasoglou et al., 2013). However, UBM is limited by its soft tissue contrast (Phoon and Turnbull, 2003), and neither UBM nor µMRI can provide the spatial resolution required for detailed phenotyping. Optical techniques expand the scale of resolution and imaging depth available for phenotyping of embryonic structures and functional features (Wang et al., 2020). Confocal, multiphoton, and light-sheet microscopy are examples of high-resolution optical microscopic methods that are capable of time-lapse imaging of early embryos in culture. However, the resulting sub-micron resolution is achieved at the expense of limited imaging depth (Lucitti et al., 2007; Larina et al., 2009b; Udan et al., 2014). Optical projection tomography (OPT) is an optical technique well suited for fluorescently labelled or label-free imaging of mouse embryos due to its relatively high resolution (5 µm) (Sharpe, 2003; Singh et al., 2016; Ban et al., 2019). However, since OPT utilizes projection images acquired at different sample orientations, which is time consuming, the imaging speed is usually sacrificed for reconstruction quality. Therefore, OPT application for live embryonic imaging is challenging. Another promising imaging technique in embryology, photoacoustic tomography (PAT), has an advantage of non-invasive fast *in utero* mouse embryonic imaging (Laufer et al., 2012; Basak et al., 2019) and allows one to measure oxygen blood saturation in addition to structural imaging (Bayer et al., 2017). However, PAT imaging is performed at significantly lower spatial resolution (Laufer et al., 2012; Bayer et al., 2017; Basak et al., 2019), which is insufficient to resolve cellular level details.

Optical coherence tomography (OCT) is a non-invasive, rapid imaging technique, which relies on natural tissue optical contrast and does not require the application of contrast agents. OCT is unique in that it offers higher spatial resolution than ultrasound or µMRI, while also having a greater imaging depth than confocal and multiphoton microscopy. With a spatial resolution of 2–10 µm and millimeter-level imaging depth (1–3 mm), the imaging capabilities of OCT are positioned between confocal microscopy and high-frequency ultrasound. Due to these specifications, OCT is uniquely suitable for mouse embryonic imaging and is capable of assessing structural dynamics in embryonic organs including the developing heart, which is only a few hundred microns in size during the early developmental stages (reviewed by (Lopez et al., 2020a)). To capture the dynamics of the embryonic hearts in 4D (3D + time), multiple approaches for direct volumetric as well as sequential data acquisition, retrospective gating, and post-acquisition synchronization have been developed (Larina et al., 2012b; Bhat et al., 2013; Wang et al., 2015b; Wang et al., 2016), which provide enhanced sampling rate and accuracy of the heartbeat reconstruction toward a time-resolved mechanistic investigation of cardiogenesis.

In addition to anatomical and morphological information, the power of OCT lies in its ability to capture functional information. One such functional extension is Doppler OCT, which detects axial blood flow velocity at the same spatiotemporal resolution as structural OCT. Doppler OCT was first established as a feasible approach to capture blood flow dynamics in the *Xenopus* embryo (Yazdanfar et al., 1997; Westphal et al., 2002; Yang et al., 2003) and with the advancement of technology, it has become an important tool to study cardiovascular development in mice (Larina et al., 2008b; Larina et al., 2009a; Gu et al., 2012; Peterson et al., 2012; Wang and Larina, 2020). Despite its advantages, Doppler OCT is not the optimal tool for vascular reconstruction due to its insensitivity to the transverse component of blood flow which prevents visualization of vessels that are perpendicular to the scanning laser beam. An alternative functional OCT approach is OCT angiography (OCTA) which highlights pixels in OCT images with higher temporal intensity fluctuations (speckle variance). Since the pixels associated with moving blood cells produce the highest fluctuations in comparison to the static tissue background, this approach allows for reconstruction of the circulatory network in live embryos regardless of the flow direction (Sudheendran et al., 2011; Kulkarni et al., 2015). This method has been extended to 4D visualization of the blood flow in the embryonic heart (Grishina et al., 2017). The cardiac 4D OCTA method relies on the periodicity of the heartbeat, and assumes an identical position of the heart wall at the same phase of each heartbeat, while the blood cells do not return to the same spatial location. 4D OCTA is performed over the frames corresponding to the same phase of the cardiac cycle, but separated in time by the whole heartbeat. By performing this analysis for each spatial location and heartbeat phase, the flow of blood cells in the heart is revealed volumetrically and dynamically. Together, these structural and functional methods provide a unique phenotyping toolset for mouse embryologists.

As outlined in this review, OCT has been applied as a non-invasive imaging tool to interrogate mouse embryo development at early stages using advanced culturing techniques (Larina et al., 2009a) (Wang et al., 2016; Wang et al., 2017), as well as at later stages when the embryo is alive and *in utero* (Syed et al., 2011; Larina et al., 2011; Larina et al., 2012c; Raghunathan et al., 2020b). A gap remains in the field with regards to integrating OCT imaging for phenotyping of transgenic and targeted mutant mice. This marriage of technology and biology has already revealed unparalleled information about the cardiac development (Lopez et al., 2015; Lopez et al., 2017) that cannot be achieved by other techniques, and the potential for its application in other developing systems is limitless. This review aims to introduce the basics of optical coherence tomography and describe its application in biology, with the intention to encourage a more cross-disciplinary approach to answering questions in development and embryology.

## Structural embryonic phenotyping

Optical coherence tomography (OCT) was first implemented in developmental biology by imaging embryonic hearts in *Xenopus-laevis* (Boppart et al., 1997). In the mouse, initial OCT investigations were performed on whole embryonic day 10.5 (E10.5) embryos, as well as extracted hearts at E14.5 and E17.5 (Luo et al., 2006). OCT was used for non-destructive optical sectioning of whole mouse embryos and the cardiovascular system, enabling visualization of embryological morphology with resolutions comparable to standard histology (Luo et al., 2006).

Jenkins and colleagues demonstrated that OCT was capable of imaging extracted, exogenously paced embryonic hearts (Jenkins et al., 2006). Developing hearts were dissected at E13.5 and placed in an electrode chamber that stabilized the heart in a temperature-controlled, oxygenated solution. The electrode chamber consisted of two platinum sheets embedded into a silicone pad which facilitated pacing of the heart at 1 Hz through field stimulation (Jenkins et al., 2006). OCT imaging of E13.5 cardiac development revealed the four-chambered structure by this time; the trabeculations of the rough-walled ventricles were discriminated from the smooth-walled atria (Jenkins et al., 2006).

**FIGURE 1:**
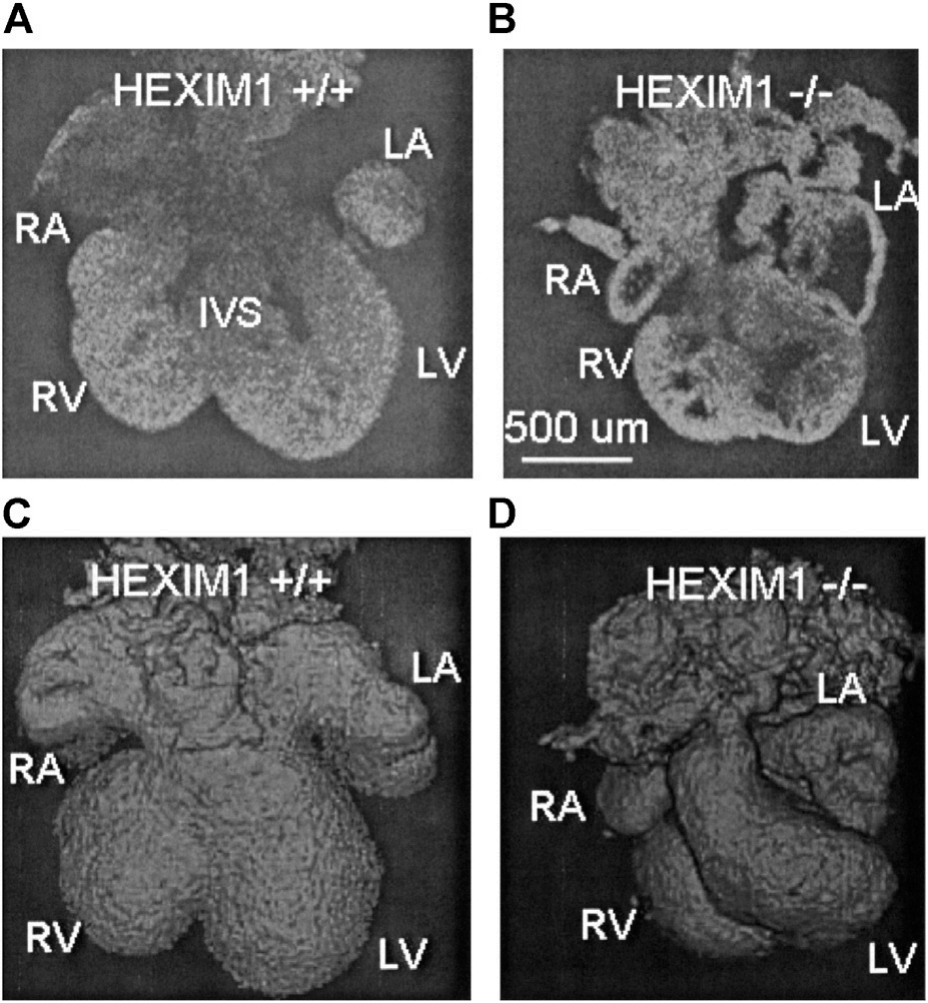
OCT structural phenotyping in Hexim1 mutant embryos. Two-dimensional (2-D) OCT images of extracted **(A)** Hexim1 wild-type and **(B)** Hexim1 mutant hearts reveal enlarged ventricular cavities and thinned myocardium in the mutant heart. Three-dimensional (3-D) volume composite images reconstructed from OCT data of **(C)** wild-type and **(D)** mutant hearts reveal that mutant hearts are smaller and developmentally delayed. RA, right atrium; RV, right ventricle; LV, left ventricle; LA, left atrium; and IVS, interventricular septum. Reproduced from (Jenkins et al., 2007).

Due to its high resolution, fast imaging speeds and non-destructive imaging capabilities, OCT quickly became recognized as a valuable phenotyping tool for mouse mutants of cardiac development. The very first OCT phenotyping of mouse embryonic hearts was performed in Hexim1 (hexamethylene-bis-acetamide-inducible protein) mutants (Jenkins et al., 2007). *Hexim1* is a tumor suppressor and a cyclic-dependent kinase inhibitor, and deletion of its C-terminal region leads to perinatal death, growth retardation, abnormal coronary patterning, ventricular wall thinning and increased ventricular cavity size (Montano et al., 2008). Hexim1 mutant and wild-type embryonic hearts were excised at E12.5 and maintained in culture to facilitate exogenous pacing at 1 Hz. 3-D data of the end-relaxation phase in the cardiac cycle revealed that mutant hearts were smaller, less developed, and asymmetrical in comparison to the wild-types. OCT-derived ventricular measurements demonstrated that Hexim1 mutant hearts showed increased ventricular chamber volumes and decreased myocardial wall thickness (Figure 1). These findings were consistent with traditional histological phenotyping techniques used to study Hexim1 mutants and demonstrate how OCT may be applied to phenotype embryonic mutant hearts in a non-destructive manner, preserving 3-D architecture and facilitating subsequent tissue processing for genotyping or histology/histochemistry.

In addition to mouse mutants of cardiogenesis, OCT has been applied for structural phenotyping of a mouse model with limb defects. Pcgf3 mutants are known to exhibit defects in bone development, but structural OCT imaging facilitated the first 3-D assessment of embryonic limb anatomy (Larina et al., 2012d). E15.5 embryos were extracted for imaging and 3-D reconstructions revealed abnormal digit patterning, decreased forelimb length as well as abnormal ankle development in Pcgf3 mutant embryos in comparison to wild-types (Larina et al., 2012d). This work highlights the necessity for non-destructive advanced imaging techniques to identify genetic factors influencing anatomical development.

OCT imaging of brain ventricular volume and blood vessel architecture demonstrate its potential as a phenotyping tool in other organ systems and mouse mutants. Specifically, the effect of maternal ethanol exposure on fetal brain development was investigated in E12.5 embryos fixed in paraformaldehyde, and OCT imaging revealed significantly increased brain ventricular volumes in the fetus (Sudheendran et al., 2013). Other studies investigating the susceptibility of fetal growth and development to maternal substance abuse have been performed using an *in utero* OCT imaging technique and will be discussed in detail later in this manuscript.

These studies demonstrate how structural OCT can be applied to generate detailed structural annotations of whole mouse embryo development, with unlimited potential regarding embryonic stage and organ system.

## Live phenotyping in culture

The first attempt at dynamic OCT mouse embryo imaging was reported by Luo and colleagues (Luo et al., 2006). The freshly dissected E10.5 embryo was imaged within the amniotic sac and OCT imaging at 5 frames per second captured cardiac structures at different phases of contraction. This study revealed the potential for cardiac phenotyping using OCT. This study also demonstrated a strong need for improved methods maintaining embryo viability during the imaging and data acquisition periods to yield physiologically relevant and informative functional data.

The integration of OCT technology with static mouse embryo culture protocols was critical for enabling imaging under semi-physiological conditions. Static embryo culture involves skilled micro-dissection under a temperature-controlled microscope station, followed by embryo recovery in an environmentally controlled incubator for at least 1 hour before imaging (Larina et al., 2012a). Modern embryonic culture systems have adapted for OCT imaging by placing the scanning head of the OCT system within a commercial tissue culture incubator, thereby maintaining physiological embryonic conditions at 37°C and 5% CO_2_, so that early embryos can be sustained (Larina et al., 2012a). Prolonged OCT time-lapse imaging of embryos in static culture can be achieved by adding rat serum to the culture medium to support embryo growth and using a thin piece of Teflon film on top of the medium to prevent evaporation (Wang et al., 2017). Early stage mouse embryos can be maintained in static culture for up to 24 h to allow for dynamic imaging of developmental processes.

This approach was applied to visualize the process of neural tube closure in mouse embryos for almost 17 h of culturing (Wang et al., 2017). The neural tube arises from the neural plate through a complex process of folding, and eventually gives rise to the spinal cord and brain. Failure of cranial neural tube closure results in anencephaly which is an absence of a major portion of the brain and results in postnatal death. In mice, cranial neural tube closure was visualized using time-lapse 3-D OCT imaging (Wang et al., 2017). At the beginning of the imaging period, E8.5 embryos had open head folds, but 13.75 h later, closure of the cranial neural tube was complete (Wang et al., 2017). OCT imaging was capable of visualizing and quantifying the zipper-like mechanism of hindbrain closure, supporting previous reports of sequential fusion of the neural folds within this region (Pyrgaki et al., 2010; Yamaguchi et al., 2011; Massarwa and Niswander, 2013). In contrast, a different mechanism of neural tube closure was identified in the midbrain region. 3-D OCT imaging revealed that the neural folds approach each other in a more simultaneous manner, suggesting a button-like closure mechanism (Wang et al., 2017). This study reports the first time-lapse 3-D OCT imaging of cranial neural tube development in the live mouse embryo within the intact yolk sac, exemplifying the ability to capture live and dynamic developmental processes upon integration of advanced imaging techniques and modular culture methods.

**FIGURE 2:**
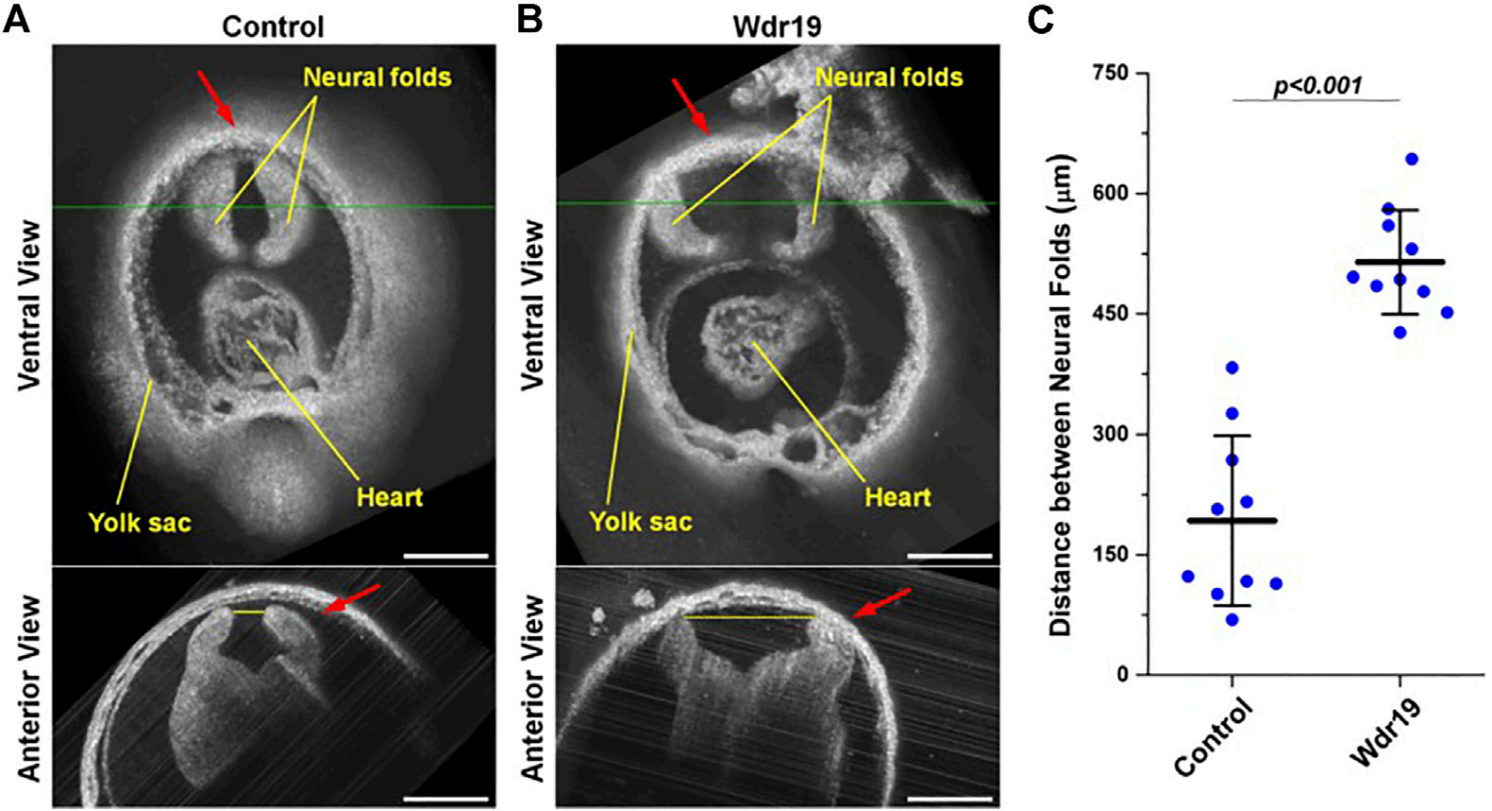
Live phenotyping in culture of the Wdr19 mutant mouse embryo. 3-D OCT images of the forebrain region from **(A)** control and **(B)** Wdr19 mutant embryos at E8.5 show an obvious difference in neural tube phenotype. The red arrows indicate the forebrain region. The green lines in the ventral views indicate the locations where the anterior views are taken. The yellow lines in the anterior views indicate the positions of distance measurement (scale bar: 300 µm). **(C)** Quantification of the distance between the neural folds at the forebrain region in control and Wdr19 mutant embryos. The distance between the neural folds is significantly larger in Wdr19 mutant embryos compared to controls (*p* < 0.001, two-sample *t* test). Data are presented as mean ± standard deviation. Reproduced from (Wang et al., 2017).

A similar approach was applied as a phenotyping tool to characterize a neural tube defect in the Wdr19 mutant mouse model (Lopez et al., 2015; Wang et al., 2017). WDR19 gene is involved in the formation and maintenance of cilia. 3-D visualizations of the head folds of control and Wdr19 mutant mouse embryos revealed differences in neural tube closure at the forebrain region (Figure 2). When quantified, Wdr19 homozygous embryos showed a significant difference in the distance between neural folds at E8.5 in comparison to control littermates, supporting a role for cilia in the process of neural tube closure (Wang et al., 2017).

The combination of static embryo culture with OCT technology provides unprecedented resolution to capture the formation and function of the cardiovascular system in mouse embryos (Larin et al., 2009). By E7.5 in mice, aggregates of cells called blood islands emerge in the developing yolk sac, which give rise to endothelial cell precursors and primitive erythroblasts (Shalaby et al., 1997; Larin et al., 2009; Garcia and Larina, 2014). A distinct feature of cardiac embryonic development is that the heart begins to contract before the advent of blood circulation, pointing towards a role for hemodynamic forces in cardiovascular development (Lopez et al., 2020a). In mice, the primitive heart tube begins to contract at E8.0, but primarily pumps plasma throughout the vascular network and embryo (Larin et al., 2009). At E8.5, primitive erythroblasts enter the circulation while the heartbeat becomes stronger. The embryonic heartbeat was first visualized with OCT by Larin and colleagues in live embryo culture using non-gated four-dimensional (4-D) reconstructions (Larin et al., 2009). The resulting hemodynamic shear force upon the vascular wall is a physical cue to induce vascular remodeling, which is a critical step in early embryonic development involving transformation of the primitive capillary plexus into a branched hierarchical network of arteries, veins and capillary beds (Lucitti et al., 2007; Culver and Dickinson, 2010). Numerous studies have shown that the physical trigger of hemodynamic force and shear stress upon the endothelium is necessary to activate vascular remodeling (reviewed by Garcia et al., 2014 (Garcia and Larina, 2014)). Despite the paucity of literature exploring these processes *in vivo*, OCT imaging of embryos maintained in static culture has led to significant advancements in this field (Wang et al., 2016; Wang and Larina, 2020).

**FIGURE 3:**
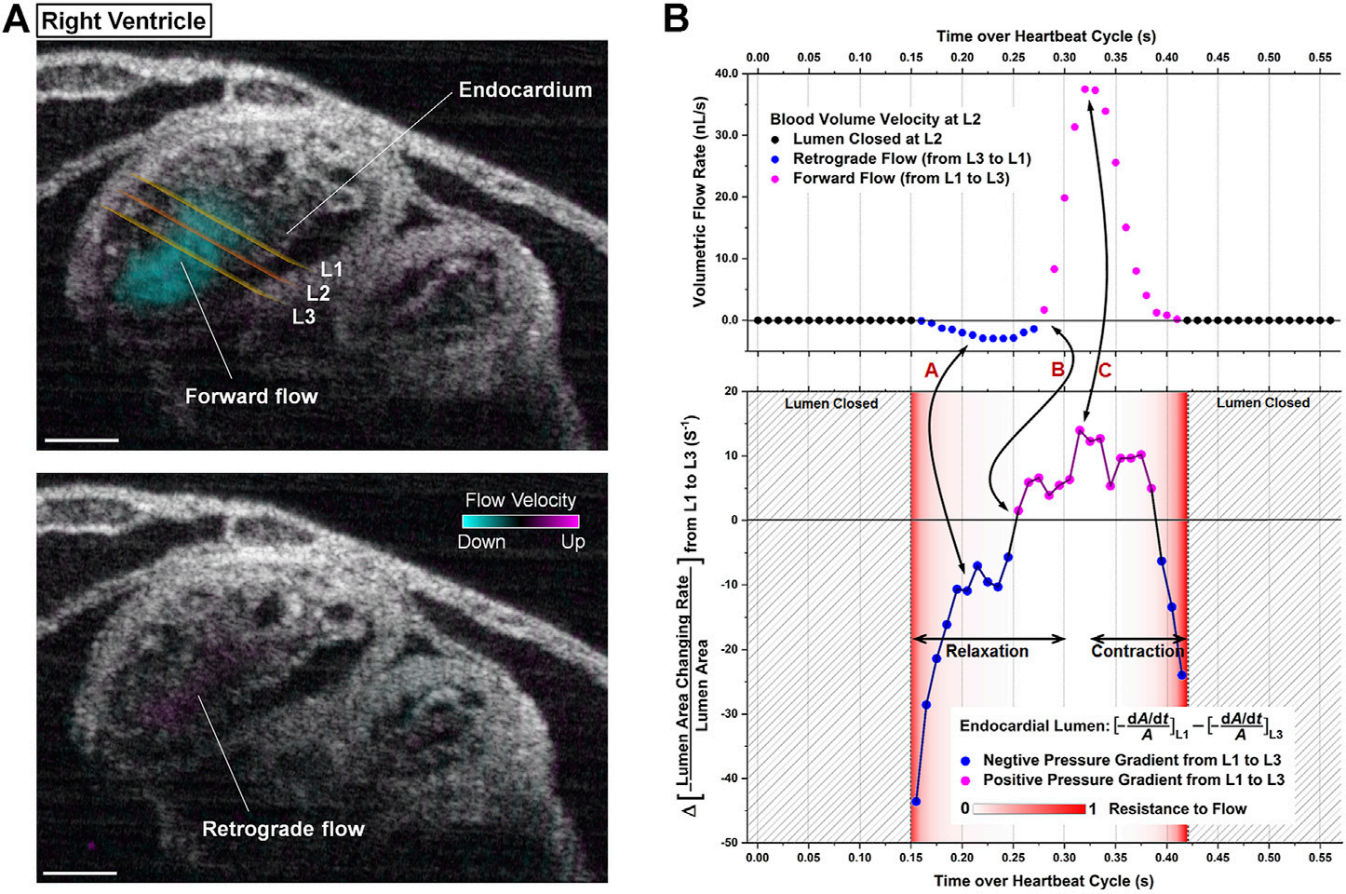
4D-OCT analysis of cardiac pumping in the right ventricle of E9.25 embryonic heart. **(A)** Structural and color-coded Doppler 4D-OCT flow images of the beating heart. The positions of the measurement planes where the analysis was performed are labeled as L1, L2, and L3. (Scale bar: 100 µm). **(B)** Corresponding quantitative analysis defining the relation between pressure gradient, resistance to flow, and volumetric flow rate toward the localized pumping investigation. Reproduced from (Wang and Larina, 2020).

Wang and colleagues achieved the first live 4-D hemodynamic imaging of the beating heart in mouse embryos and demonstrated that this approach is capable of providing high resolution visualization and quantification of blood flow profiles (Wang et al., 2016) and cardiac pumping dynamics (Wang and Larina, 2020). This work revealed the first imaging and analysis of retrograde flows through the mouse embryonic heart, which have previously been demonstrated in zebrafish (Liebling et al., 2006; Vermot et al., 2009) and avian (Midgett et al., 2015) models and suggest the influence of shear stress on the cardiac wall, thus supporting a role for hemodynamic forces and biomechanical stimuli during development (Wang et al., 2016). Cardiac pumping was assessed based on the relation between blood flow rate, blood flow resistance and the pressure gradient, which revealed connections between the temporal profiles of pressure gradient and volumetric blood flow rate (Wang and Larina, 2020). This work describes an active pumping mechanism in the early embryonic ventricles which involves a combination of suction and pushing (Figure 3) (Wang and Larina, 2020). The application of 4-D hemodynamic OCT in this study, highlights the richness of data that can be extrapolated, and reveals the potential for investigating the regional relation between blood flow and heart wall dynamics which will be critical for improving the interpretation of hemodynamics in mouse mutants modeling human congenital heart defects at early embryonic stages.

The structural and dynamic cardiac imaging integrating OCT with static embryo culture was implemented for mouse embryo cardiac phenotyping. The first application was in Wdr19 (WD repeat-containing protein 19) mutants (Lopez et al., 2015). WDR19 is involved in the formation and maintenance of cilia, and its mutations have been found in human patients with ciliopathies such as cranioectodermal dysplasia which is characterized by skeletal, ectodermal, connective tissue, renal and liver anomalies (Lin et al., 2013). In addition to defective neural tube closure, E8.5 Wdr19 mutant embryos displayed abnormal heart tube morphology compared to control embryos. Cardiac looping, which is the rightward bending and rotation of the primary heart tube, was defective in Wdr19 mutants whereby the looping angle in the mutant hearts was significantly smaller compared to controls (Lopez et al., 2015). Cardiac looping is required for proper alignment of the ventricular chambers with the outflow vessels and atria, and therefore, Lopez and colleagues used OCT to visualize early embryonic cardiodynamics in mutant embryos by performing B-scan imaging over time at different locations throughout the heart. Subsequent data acquisition and synchronization resulted in the reconstruction of the heart wall movement and blood circulation. Despite the obvious looping defect, the heart rate of the mutant embryos was in the normal range suggesting that the primary cardiac defect was structural rather than functional (Lopez et al., 2015). This work exemplifies the advantages of using dynamic OCT cardiac imaging in combination with embryo culture for live phenotyping of early embryonic cardiac morphogenesis facilitating structural and quantitative cardiac phenotyping.

4-D OCT cardiodynamic imaging was also implemented as a phenotyping tool for Mlc2a (atrium specific myosin light chain 2) mutant embryos. *Mlc2a* is expressed in embryonic hearts from the initiation of contraction, and its deletion regionally affects contractility (Sheikh et al., 2015; Lopez et al., 2017). Deletion of Mlc2a leads to embryonic death at E10.5, with hearts undergoing edema and failure (Lopez et al., 2017). Using Doppler OCT, dynamic blood flow imaging was performed in early embryonic mouse hearts (E9.0) and was correlated with heart wall movement to reveal significantly reduced contractility in Mlc2a mutant hearts compared to controls, highlighting the capability of OCT phenotyping to capture not only structural defects, but also functional deficiencies in genetic mouse models (Lopez et al., 2017).

Integrated OCT imaging and mouse embryo culture systems have also been shown to assess blood profiles (Larina et al., 2008b), the velocity of individually circulating blood cells (Larina et al., 2009a), and vascular remodeling of the embryonic yolk sac (Sudheendran et al., 2011), all of which can potentially be used to interrogate structural and functional cardiovascular defects in mutant mice.

## *In utero* imaging

The major limitation of embryonic OCT imaging in static culture described above is that the early embryos can only be cultured for 24–48 h, and embryos beyond the E10.5 stage do not survive in culture for prolonged time. In order to capture long-term developmental processes in the same embryo, a method for live imaging of embryos *in utero* has been developed which involves externalization of the uterine horn through an abdominal incision. This approach is appropriate for embryos beginning at E12.5 until the remainder of gestation. It is challenging to image embryos earlier than E12.5 *in utero* because of the thick decidual layer that surrounds each embryo which attenuates the signal. As the embryos grow, this layer becomes thinner and degenerates by E12.5 allowing for imaging through the uterine wall that also stretches and thins as development progresses. By sequential exposures, this method allows for repeated imaging of the same embryos *in utero* over different developmental stages with unprecedented resolution (Larina et al., 2011). *In utero* OCT imaging not only allows for the visualization of developing embryonic structures in real time, but also enables phenotypic characterization of developmental timelines in the limbs (Syed et al., 2011), eye (Larina et al., 2012c) and brain (Syed et al., 2011).

**FIGURE 4:**
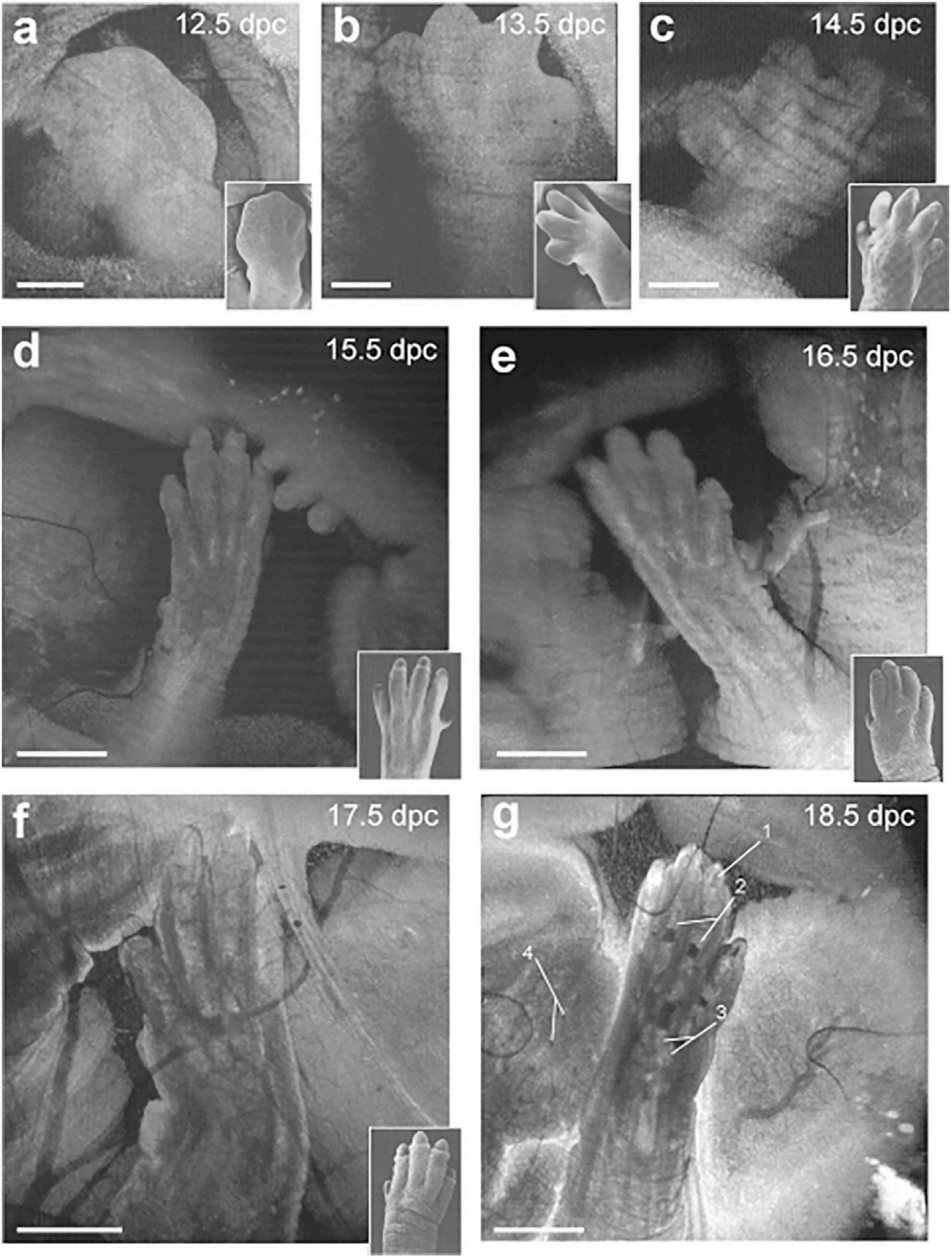
Live *in utero* phenotyping of mouse embryo limb development. **(A–C)** 3-D reconstructions of embryonic forelimb development at E12.5, E13.5 and E14.5 (scale bar: 500 µm). **(D–G)** 3-D reconstructions of digit formation at E15.5, E16.5, E17.5 and E18.5 (scale bar: 1 mm). Insets show scanning electron micrographs of the embryonic forelimb at the corresponding embryonic stages 1, cartilage primordium of distal phalanx; 2. Cartilage primordium of proximal phalanx; 3, cartilage primordium of metacarpal bones; 4, follicles of vibrissae. Reproduced from (Syed et al., 2011).

Toward investigation of limb development, *in utero* OCT can be used to depict morphological changes from E12.5 to E18.5 (Figures 4A–C) (Syed et al., 2011). The process of digit formation involves indentation of the interdigital zones, digital splaying and elongation, claw development, as well as programmed cell death of interdigital webbing (Figures 4D–G). Each of these milestones were visualized by OCT, therefore allowing specific phenotypes to be characterized at discrete developmental stages of the limbs (Syed et al., 2011). Furthermore, it was possible to distinguish the point of cartilage formation in the developing forelimb bones. OCT images at E13.5 showed digits that were uniform in structure, however, OCT scans demonstrated non-uniform structure of the digits and palms at E17.5 due to the appearance of the cartilage primordium (Figures 4F,G) (Syed et al., 2011). It is clear from this work that *in utero* OCT imaging is not only capable of characterizing the morphological milestones of limb development, but also provides detailed contrast of bone and cartilage (Figure 4G).

To date, *in utero* OCT has not been applied as a phenotyping tool for models of limb abnormalities, but it has the potential to replace standard phenotyping techniques such as alcian blue alizarine red staining, which is both time-consuming and laborious; and cannot be applied in live embryos (Glaser et al., 2017). By contrast, the method described here would circumvent the need for processing multiple litters and would allow for high-throughput analysis for use in large-scale genetic screens. Furthermore, it could be implemented longitudinally to determine the timeline of defect progression in mutants exhibiting phenotypic variability.

This potential is further supported by using *in utero* OCT to study embryonic ocular structure at three different stages and thus, allowing for key morphological landmarks of eye development to be appreciated (Larina et al., 2012c; Sudheendran et al., 2015). At E13.5, the lens is relatively small, the cornea has formed and there is evidence of eyelid differentiation. By E15.5, the lens is larger in size, the anterior chamber of the eye has formed and the hyaloid vasculature is evident. At E17.5, all structures have enlarged and the conjunctival sac has formed (Sudheendran et al., 2015). Throughout the same stages of development, *in utero* quantification of ocular structures can accurately assess the increasing diameter and volume of the eye lens and globe that accompanies ocular growth (Sudheendran et al., 2015). Overall, the optical contrast afforded by OCT imaging allows for the differentiation of ocular structures so that developmental stages are distinguishable. Furthermore, *in utero* OCT imaging at E16.5 is suitable for visualization of the hyaloid capillary network, which is the primary intraocular vascular network that regresses later in development to ensure optical transparency for proper vision (Larina et al., 2012c). Failure of the hyaloid vasculature to regress results in retinal detachment, cataracts and glaucoma, and the imaging approach described by Larina and colleagues might represent a new avenue to explore this disorder in mouse models (Larina et al., 2012c). However, other ocular abnormalities, such as tumor development, have been explored *in utero* with OCT imaging. Larina and colleagues performed the first non-histological structural characterization of the retinoblastoma mouse model, Pax6-SV40 T-antigen, which spontaneously forms lens and retinal tumors during development. The ocular lens of transgenic embryos at E15.5 was highly scattering and appeared brighter than that of wild-type littermates due to the developing tumor, therefore facilitating unambiguous differentiation of transgenic and wild-type embryos *in utero* (Larina et al., 2012c). These studies demonstrate that OCT imaging *in utero* not only provides dynamic information about embryonic morphogenesis, but also highlights its potential in studying the progression of diseases.

**FIGURE 5:**
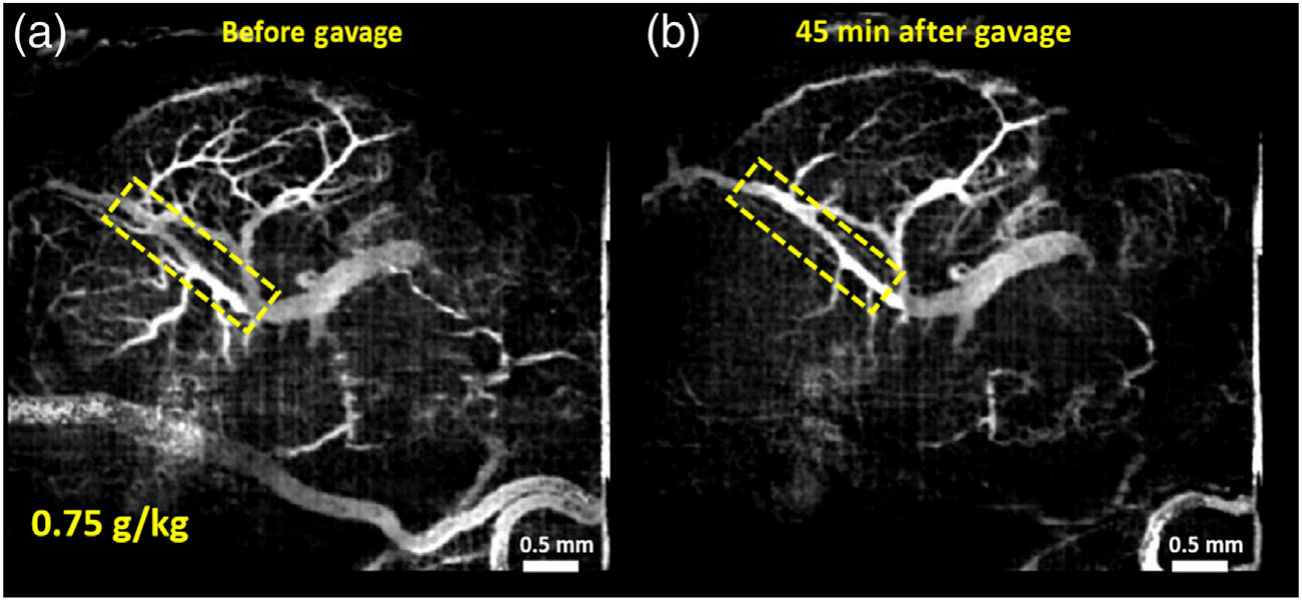
Maximum intensity projection (MIP) of correlation mapping optical coherence angiography (cm-OCA) of murine fetal brain vasculature. **(A)** MIP cm-OCA of fetal brain vasculature before maternal exposure to ethanol. **(B)** MIP cm-OCA of fetal brain vasculature 45 min after maternal exposure to ethanol at a dose of 0.75 g/kg showing acute vasoconstriction. The dashed rectangle shows the main branch of the vessel on which quantifications were performed demonstrating the effect of ethanol. Reproduced from (Raghunathan et al., 2020b).

Another application includes *in utero* structural imaging of the embryonic brain, its vasculature, and the prenatal effects of pharmacological and toxicological agents (Raghunathan et al., 2018; Raghunathan et al., 2019; Raghunathan et al., 2020a; Raghunathan et al., 2020b). Using the same method of OCT imaging *in utero*, it is possible to investigate the cerebral cortex and brain ventricles from E13.5 to E16.5, while after E16.5, as the skin and skull develop, the ventricles become inaccessible for optical imaging but the cortex remains within the imaging depth (Syed et al., 2011). The embryonic brain microvasculature can also be delineated using functional OCT, and OCTA is capable of capturing the dynamic changes in vessel diameter in response to maternal teratogen exposure (Raghunathan et al., 2018; Raghunathan et al., 2019; Raghunathan et al., 2020a; Raghunathan et al., 2020b). The microvasculature that develops in the fetal brain during the 1^st^ and 2^nd^ trimester supports nutritional needs, provides endocrine control of fetal growth, and promotes neural development (Raghunathan et al., 2018). *In utero* OCTA imaging (also called optical coherence angiography, OCA) was applied to evaluate the brain microvasculature in mouse embryos at E14.5 in response to maternal consumption of alcohol in a binge-like setting (Raghunathan et al., 2018), as well as in a dose-response manner (Raghunathan et al., 2020b). Raghunathan and colleagues show a dramatic decrease in vessel diameter 45 min after binge-like maternal alcohol exposure which did not happen in the sham group where changes in vessel diameter over time were minimal (Raghunathan et al., 2018). There was a dose-dependent reduction in vascular diameter in fetal brain vessels when the mother was exposed to ethanol (Figure 5) and simultaneous imaging of the maternal peripheral vessels showed that hindlimb vessels exhibited concurrent vasodilation (Raghunathan et al., 2020b). These studies demonstrate that maternal alcohol consumption can cause drastic vasoconstriction during the critical period of neurogenesis and angiogenesis, which could significantly diminish nutritional supply and have permanent detrimental effects on fetal brain development.

Prenatal exposure to nicotine (Raghunathan et al., 2020a) and cannabinoids (Raghunathan et al., 2019) was also assessed in mouse embryos during the second trimester-equivalent period of gestation (E14.5). Fetal brain vasculature was notably decreased following maternal exposure to both teratogens (Raghunathan et al., 2019; Raghunathan et al., 2020a). *In utero* OCTA imaging revealed constriction of brain vasculature, a loss of branching vessels, as well as decreased vessel diameter, length fraction and area density. Similar to prenatal exposure to alcohol, these studies show that fetal brain vasculature undergoes significant vasoconstriction following acute prenatal exposure to nicotine and cannabinoids during a critical window of gestational development (Raghunathan et al., 2019; Raghunathan et al., 2020a). Taken together, these studies showcase the unique opportunity to study embryonic vasculature *in utero* using OCT and pave the way for future phenotypic investigations of a variety of mutant models, including the assessment of teratogens on cardiac vasculature development.

The studies outlined above have all been performed by externalization of the uterine horn through an abdominal wall incision. Methods for intravital imaging are being developed which allow for prolonged and longitudinal embryonic imaging with fluorescence microscopy (Huang et al., 2020), as well as OCT imaging of the mouse reproductive system (Wang et al., 2015a; Wang and Larina, 2021; Umezu et al., 2022). Such methods could potentially be optimized for prolonged and functional embryonic phenotyping with OCT.

## Summary and future perspectives

This review is intended as a reference for developmental biologists and embryologists interested in exploring the applications of OCT in the context of mouse embryonic phenotyping, and aims to inspire more interdisciplinary collaborations and investigations. Here we introduce the basics of OCT technology and its approaches, and provide a detailed account of how the technology is currently being applied within the field of developmental biology, paying particular attention to mouse embryonic development and phenotyping.

Structural OCT is especially useful for generating detailed anatomical atlases of whole mouse embryo development and embryonic cardiac phenotyping (Jenkins et al., 2006; Jenkins et al., 2007; Larina et al., 2012d; Choi et al., 2020). OCT allows for non-destructive phenotyping which preserves the 3-D architecture and facilitates subsequent tissue processing for further genotyping or histology/histochemistry. The combination of static embryo culture with OCT technology has greatly expanded its imaging potential for embryonic investigations. This approach has enabled visualization and quantification of dynamic, early-embryonic events such as neural tube closure (Wang et al., 2017) and the first embryonic heartbeat (Larin et al., 2009), as well as, cardiodynamic phenotyping of mouse mutant models (Lopez et al., 2015). A critical aspect in realizing the full potential of OCT, is extending embryo culture techniques beyond 48 h and supporting mouse embryo growth in culture after E10.5. Culture conditions are continuously being improved, but innovative culture approaches will be key to visualizing early post-implantation development in culture. Roller culture provides the optimal conditions for *ex utero* embryonic growth because the embryos are maintained in constant motion, in a controlled temperature and gas environment, throughout the culture period (Piliszek et al., 2011). Roller culture, which will support 72 h of embryonic growth, is not optimal for live time-lapse imaging due to the fact that the embryos are in constant motion. However, an integrated roller and static culturing approach, by performing static culture OCT imaging at various time points over the roller culture period, could be readily incorporated into current imaging protocols. In particular, early embryonic cardiac developmental investigations will benefit from extended imaging and culturing periods, and when combined with reporter-expressing strains of genetically modified mice, will provide a powerful approach to image and phenotype live, dynamic cell behaviors *in situ*.

Later developmental events are well visualized *in utero* through externalization of uterine horns in anesthetized females which allows for repeated imaging of the same embryos (Syed et al., 2011). *In utero* OCT imaging enables visualization of developing embryonic structures in real-time with unprecedented resolution (Syed et al., 2011), allows for phenotypic characterization of developmental timelines (Larina et al., 2012c), and facilitates assessment of the prenatal effects of pharmacological and toxicological agents (Raghunathan et al., 2018; Raghunathan et al., 2020a; Raghunathan et al., 2020b).

While providing a superior volumetric imaging capability for developing mouse embryos at a micro-scale level, as with all imaging modalities, OCT is associated with some limitations. One of the limitations is imaging depth, which is restricted to approximately 1–2 mm in embryos. This is sufficient to resolve structures in cultured early stage embryos, but only allows for imaging of external layers at later stages and *in utero* (Syed et al., 2011). Optical clearing has been shown to enhance the OCT imaging depth in embryonic tissues (Larina et al., 2008a). While current optical clearing methods are not compatible with live imaging, the field is actively developing. Optical clearing was previously applied *in vivo* in mice for enhanced visualization of the brain vasculature (Feng et al., 2019), which suggests a potential for its application in embryo imaging. Alternatively, tomographic approaches such as rotational imaging OCT (RI-OCT) can be implemented to partially overcome the imaging depth limitation. RI-OCT was demonstrated to reveal the complete 3-D structure of E9.5 and E10.5 mouse embryos by performing conventional 3-D OCT imaging at four different angles (Chen et al., 2016). Another limitation of OCT imaging is a lack of contrast between cells within tissue layers. OCT relies on natural tissue contrast for image construction, which is a major advantage of the technology; however, the imaging is limited to the endogenous contrast. Contrast agents for enhanced OCT imaging are currently being developed. Beyond embryology and developmental biology, exogenous contrast agents such as gold nanoshells are being explored for OCT image enhancements (Si et al., 2018). For example, Ring et al. recently demonstrated an enhanced *in vivo* detection of skin pathology using clinical OCT (Ring et al., 2019). Genetically encoded OCT contrast are also being developed (Lu et al., 2020). Further development of these technologies will significantly enhance the capability of OCT for imaging cell migrations, lineage tracing, and studying cell differentiation.

Multimodal imaging is an attractive approach for embryonic phenotyping because of the diversity of developmental processes and the fact that a single type of imaging contrast can only provide limited information. The area of OCT embryonic phenotyping has great potential to benefit from the implementation of multimodality approaches, where each technology is providing unique complementary information. Structural OCT has previously been used in combination with spectrally encoded confocal microscopy (Yelin et al., 2007), Brillouin microscopy (measures tissue stiffness) (Raghunathan et al., 2017; Zhang et al., 2019), and photoacoustic tomography (Liu et al., 2014) to provide a comprehensive picture of the morphological and dynamic events for other biological applications. Wu and colleagues explored the complementarity of selective plane illumination microscopy and rotational imaging optical coherence tomography toward embryonic structural analysis (Wu et al., 2017). Second harmonic generation microscopy provides well-defined imaging contrast for fibrillar proteins such as collagen without labeling (Lopez and Larina, 2019). Integrating OCT with this technology would potentially enhance biomechanical analysis of embryonic development. The field will also benefit from advancements in other OCT-based biomechanical imaging, such as OCT-elastography, which measures tissue elasticity (Wang and Larin, 2015). OCT can also be integrated with embryonic manipulation techniques for OCT-guided micro-injections (Syed et al., 2015), as well as optogenetic techniques for OCT imaging of optogenetic cardiac pacing in mouse embryos (Lopez et al., 2020b). With the continued advancement of technology, the potential for embryonic phenotyping will also expand.

In summary, OCT technology provides a unique toolset for structural and functional phenotyping of various aspects of embryonic development. We hope this review will inspire developmental biologists to expand their use of OCT imaging in their research. We also hope to encourage more biophotonic and optical engineers to lend their knowledge and expertise to the developmental biology community. The growing accessibility and relatively low cost of customizing OCT systems mean it will grow in popularity amongst developmental biologists as a viable and attractive approach to asking new questions about their experimental models, expanding the range of biological inquiry and promoting the development of multidisciplinary studies.
